# First Report of the Thermophilic *Thalassoma Pavo* (Linnaeus, 1758) on the Central Adriatic Coast of Italy, in Abruzzo

**DOI:** 10.3390/biology13120987

**Published:** 2024-11-29

**Authors:** Alessio Arbuatti, Alessandra Di Serafino, Pia Lucidi

**Affiliations:** 1 Department of Veterinary Medicine, University of Teramo, 64100 Teramo, Italy; aarbuatti@unite.it; 2 Center of Advanced Studies and Technology (CAST), University “G. d’ Annunzio”, 66100 Chieti, Italy; alessandra.diserafino@unich.it; 3 Department of BioSciences and Technology for Food, Agriculture, and Environment, University of Teramo, 64100 Teramo, Italy

**Keywords:** climate fish, Adriatic biodiversity, Mediterranean Sea, Trabocchi Coast, Adriatic Sea meridionalization, ornate wrasse

## Abstract

The Trabocchi Coast in the Chieti district of the mid-Adriatic (Italy) is one of the few rocky areas within the General Fisheries Commission GSA 17, alongside Mount Conero (Ancona 43°00′01″ N 13°52′13″ E) and the small San Nicola Rock (Ascoli Piceno; 43°32′0″ N 13°36′0″ E). This coastline is known for its biodiversity-rich bays, inlets, and submerged cliffs. Since 2015, annual biodiversity surveys have been conducted in the area, focusing on marine species richness and the identification of non-native species. In September 2024, a juvenile ornate wrasse (*Thalassoma pavo*) was documented for the first time in the middle Adriatic during an underwater visual survey at Trabocco Punta Torre, a key site along the Trabocchi Coast near artificial and biogenic reefs. This record extends the known distribution of *T. pavo*, a thermophilic species previously reported only along the southern Adriatic coast of Puglia. This is the first confirmed sighting on the middle and northern Adriatic coast of Italy. The discovery highlights the importance of ongoing biodiversity monitoring to track changes in marine ecosystems, particularly as the Adriatic Sea faces environmental shifts linked to climate warming. The presence of *T. pavo* in this area suggests the potential for the species to establish populations in previously uninhabited northern regions. Further research is needed to explore the role of biotic and abiotic factors—such as water temperature, current patterns, and habitat availability—in the survival and potential reproduction of *T. pavo* in the middle Adriatic. The observation contributes to the broader understanding of the meridionalization process in the Adriatic Sea, where rising water temperatures are facilitating the northward expansion of thermophilic species. Continuous monitoring is recommended to assess the long-term viability of *T. pavo* populations in the Adriatic Sea and better predict the impacts of ongoing climate change on marine biodiversity.

## Simple Summary

Climate change and rising sea temperatures are facilitating the spread of non-native and exotic species. In this context, thermophilic fish species are finding favorable conditions for their natural habitat and settlement, serving as indicators of climate change. One such species, the thermophilic *Thalassoma pavo* (commonly referred to as a “climate fish”), has historically not been found along the western (Italian) Adriatic coasts, north of the Apulia region. Here, we report the discovery of a live specimen in the shallow waters of the Trabocchi Coast in Abruzzo, a region where it had never been documented despite years of biodiversity surveys in the area. This finding contributes to the understanding of how climate change is altering marine ecosystems in the Adriatic Sea.

## 1. Introduction

*Thalassoma pavo* inhabits a broad coastal range from Cape Lopez (Gabon, Africa) to Portugal, including São Tomé, Annobon, the Canary Islands, Madeira, and the Azores [1]. Over the last quarter of the 20th century, this thermophilic species [2,3,4] extended its range into the warmer southern sectors of the Mediterranean Sea, becoming increasingly common [2,5]. The first recording of adult specimens in the northern Mediterranean Sea occurred in 1988 in western Corsica (Scandola, France), followed by juveniles in 1991 [6].

The species then reached the Italian coasts, colonizing the Tyrrhenian Sea from south to north, with reports from Ustica, the Aeolian archipelago, Ischia, Santo Stefano Isle, and the Tuscany archipelago and up to the natural reserve of Calafuria [2,3,7,8,9]. The northernmost range of the ornate wrasse includes the Ligurian Sea, with sightings from Gallinaria Island, Portofino, La Spezia, Capo Mortola, Capo Venere, and Bergeggi [3,5,10,11,12]. In the Ligurian Sea, the species has been observed reproducing, independently of the larval supply from the Tyrrhenian Sea [7,11]. In the Ionian Sea, *T. pavo* has been reported from eastern Sicily (Ciclopi Islands) up to Puglia [3], in geographical subareas GSA 19 and 18. In the central and northern Adriatic (GSA 17), the species has been recorded exclusively along the Croatian coast: Lukrum, Korčula Island, Rogoznica (Svilan Islet), the Primosten area (the Grbavak, Lukovnjak, and Maslinovik islets), Makarska, Dubrovnik, and Split [13,14,15,16,17]. The northernmost specimen was found at Cape Kosàka (Island Sveti Grgur) [18].

In contrast, *T. pavo* has only been recorded on the Italian Adriatic coast of Apulia (GSA 18), with reports from Torre Guaceto, Torre del Serpe, Torre Minervino, Zinzulusa, and Ciolo [3,19], and it was mentioned in a technical report on the fauna of the Tremiti Islands Marine Protected Area, Apulia [20]. A review of the Global Biodiversity Information Facility database [21,22] reveals that most Italian records of *T. pavo* come from the Tyrrhenian, Ionian, and Ligurian Seas, with only eight from the Adriatic, all from southern Apulia. To the best of our knowledge, this study provides the first evidence of *T. pavo* from the Italian GSA 17 zone, representing the northernmost living specimen reported from the Italian Adriatic coast.

## 2. Materials and Methods

The Trabocchi Coast, which takes its name from the ancient fishing machines along the coastal stretch in the province of Chieti (CH), Italy, overlooks the Adriatic Sea for over 50 km between the Foro and Trigno river mouths. The coast comprises an extremely varied landscape: sandy beaches alternate with inlets reachable only by sea, rocky cliffs with rare coastal dunes, and a rich variety of Mediterranean vegetation. Along the coast, there are many artificial barriers and submerged cliffs that, over time, have become home to rich animal biocenoses and algal communities, making the Trabocchi strip one of the richest areas in the central Adriatic in terms of biodiversity [23].

**Figure 1:**
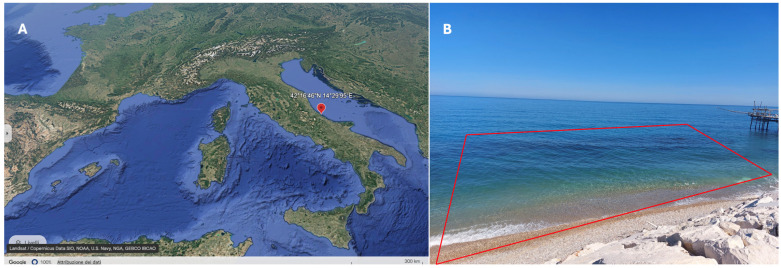
Research area (**A**), located along the Trabocchi Coast of Chieti, in the mid-Adriatic Sea (Google Earth). (**B**) The submerged cliffs in the trapezoidal area, near Trabocco “Punta Torre”, part of which is noticeable on the right end of the picture. The area—although just a few meters from the shoreline—reaches a depth of more than 5 m (Photo credit A. Arbuatti).

This study utilized the underwater visual survey (UVS) method through snorkeling and freediving along a segment of the Trabocchi Coast. The survey was conducted at a specific site, “Trabocco Punta Torre” in Rocca San Giovanni (42°16′46″ N 14°29′95″ E, Figure 1A). These assessments aimed to evaluate the qualitative biodiversity of marine species along this section of the Trabocchi Coast using a non-destructive approach.

The study area where the fish has been signaled is part of the Trabocchi Coast that we have monitored since 2015. It has a trapezoidal shape and is approximately 72 m long at the side of the submerged cliffs (16 m wide), with a more extended shoreline base, yielding a total surveyed area of about 3300 m^2^ (Figure 1B). A GoPro Hero 11 (GoPro GmbH, Baierbrunner Str. 15 Bldg. D, 81379 Munich, Germany; 4K 60 fps) with a waterproof case was used to witness the richness of Trabocchi’s marine life. The UVS always took place in the morning under natural light conditions, taking advantage of the sea’s relative calm.

## 3. Results

On 7 September 2024, a specimen of the ornate wrasse (*Thalassoma pavo*) was recorded for approximately 2 min and 30 s. The observation occurred during a morning UVS session (10:50 a.m.) and focused on an artificial and biogenic reef located 40 m from the shoreline. The water temperature at the time of the survey was recorded at 27 °C.

**Figure 2:**
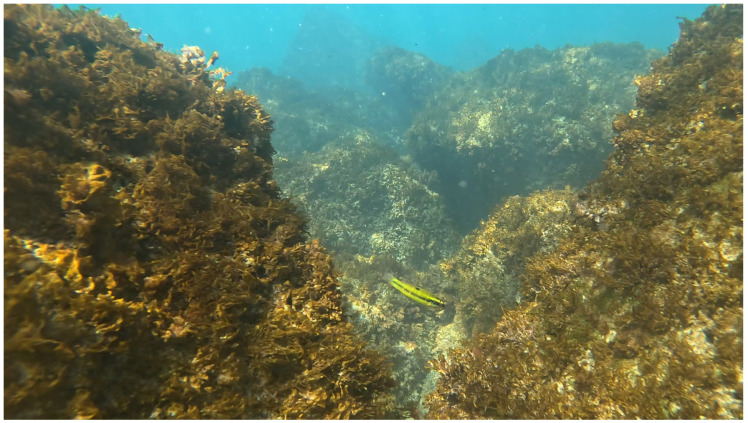
Picture of a *T. pavo* specimen swimming among the rocks on the Trabocchi seabed, recorded during a session of an underwater visual survey taken under natural light conditions and free diving in September 2024 (photo credit: A. Arbuatti).

**Figure 3:**
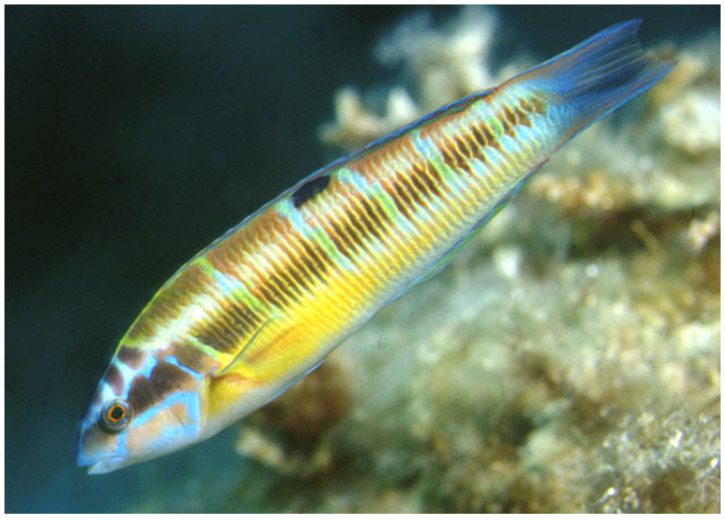
A *T. pavo* specimen from Baleares (Spain, 1988) included in the FishBase, database [25] a global information system on fish (photo courtesy of R.A. Patzner).

*Thalassoma pavo* is a thermophilic, protogynous labrid species known for its distinctive color patterns, which change with life cycle stages [24]. The recorded specimen exhibited an intermediate transitional livery between the juvenile yellow/green color pattern, including the characteristic dark spot at the base of the dorsal fin and the fragmented longitudinal dark band associated with adult stages (Figure 2). Figure 3 shows a specimen of *T. pavo* from a well-known marine database [25] for comparison with the specimen from the Trabocchi Coast.

Upon the magnification of the images, typical light blue sub-jugular reflexes were visible. The individual was estimated to be approximately 8 cm long, appeared healthy, and was observed actively swimming and foraging among the reef’s cracks. The fish was primarily located in the well-lit, shallow portion of the submerged reef at a depth of around one meter, but it was also seen moving toward the external base of the reef at a depth of 3.2 m.

## 4. Discussion

*Thalassoma pavo* is a thermophilic species typically found along the coasts of Italian seas, except for the middle and upper Adriatic Sea [26]. Of the ten years of UVS summer observations along the Trabocchi Coast, this is the first time a specimen of *T. pavo* has been recorded. Although the observation of a single individual does not necessarily indicate an ongoing population establishment, this sighting marks the first documented occurrence of the species along the central and northern Adriatic coasts of Italy. It indicates that *T. pavo* is capable of surviving in this region.

Understanding the origin of this *T. pavo* specimen requires a consideration of various biotic and abiotic factors that could influence the species’s potential future establishment on the western (Italian) coast of the Middle Adriatic Sea. Firstly, *T. pavo* inhabits shallow rocky environments [2], where coralligenous reefs, animal biocenoses, and algal communities are well developed [14]. In this context, the Trabocchi Coast is an important monitoring site in the central western Adriatic, rich in rocky shorelines, bays, inlets, and submerged cliffs. The local ecosystem is considered one of the most biodiverse in the Adriatic [23]. Notably, within the General Fisheries Commission for the Mediterranean’s Geographical Sub-Area 17, the only natural rocky portion of the Italian Adriatic, apart from Mount Conero (Ancona) and the little San Nicola Rock (Ascoli Piceno) (ADRIREEF [27]), pertains to the Trabocchi Coast, forming a unique habitat for fish that thrive in shallow coastal waters. Moreover, compared to the northwestern Adriatic, which is more affected by river runoff and higher primary productivity, the central Adriatic experiences lower productivity, which may explain differences in habitat suitability [28].

Secondly, the Adriatic Sea’s general surface circulation follows a large-scale cyclonic pattern [28]. The Eastern Southern Adriatic Current (ESAC) enters from the Strait of Otranto and flows along the eastern Adriatic before turning southward along the Western Adriatic Coastal Current (WACC) [29,30,31]. Additionally, three marine gyres operate within this system, including the Middle Adriatic Gyre (MAG), originating from the Jabuka/Pomo Pit, which circulates counterclockwise between Croatia and Abruzzo [32,33,34]. Although with specific seasonality, the Trabocchi coast of Abruzzo is therefore crossed by the WAAC but could also receive part of the water current from the Pomo circular flow. It would be interesting to investigate whether these currents (WACC and MAG) could have transported floating eggs, larvae, and other biological matter from the eastern Adriatic towards the Trabocchi Coast.

While it is somewhat speculative to sustain that WAAC could have transported eggs or larval forms of *T. pavo* from the north Adriatic (there are no reports of the presence of the species further north of Abruzzo in the Italian side of GSA17), the provenience of specimens from the mid-eastern (or southeastern) Adriatic through MAG could not be ruled out. According to Falco and colleagues [35], the transport of passive tracers that entered through the Strait of Otranto has resulted in different trajectories, one of these being the recirculation in the central Adriatic subbasin for up to two months. The circulation of surface waters in the Jabuka/Pomo pit is indeed directed in a counterclockwise path and, as in the entire Adriatic, is composed of waters that come from the south, go up the Croatian coasts, and go back south on the Italian part [32,34,36]. Although theoretical, it cannot be excluded that larval forms of *T. pavo* could have been transported by these currents from the eastern Adriatic. On the other hand, instead, the northwestward ascent of eggs, larvae, or fish from the Apulian coasts should be less likely, given that, unlike the eastern Adriatic, where a northward expansion via the ESAC is plausible, the WAAC system could inhibit the movement from south to north. However, if more specimens were found, further research should be carried out to determine whether these populations are “true” reproductive populations or dependent on larval input from other regions, as observed in Liguria [2].

*Thalassoma pavo* is an oviparous, protogynous hermaphrodite that reproduces in spring and summer [37]. In the Ligurian Sea, *T. pavo* spawning has been reported to occur when the sea surface temperature reaches 23 °C (late June), but it can last until September or, at least, when the photoperiod drops below 12 h [11]. Planktonic eggs and larvae distribute at depths of up to 50 m [36]. The Planktonic Larval Duration (PLD) for *T. pavo* has been estimated to last 38–49 days by Raventós and Macpherson [38], though durations can vary significantly, with tropical species of the genus *Thalassoma* having PLDs exceeding two months [39,40]. Wrasse larvae are known to disperse over vast distances, sometimes up to 1200 km offshore [40,41], confirming the species’ potential for long-distance larval dispersal. The specimen observed on the Trabocchi reef measured approximately 8 cm in length, placing it within an intermediate size class as defined by Guidetti et al. [2]. Given its size and livery, it likely hatched in 2023. Suppose the spawning period in the Adriatic coincides with that recorded in the Ligurian Sea. In that case, two hypotheses arise: either the fish arrived in this season as a partially developed juvenile, carried via the currents, or it has been present in juvenile form for some time, having arrived last year as a larva, survived the winter (avoiding the most challenging period in autumn), thanks to the mild temperatures in the Adriatic [42], and finally been observed in September 2024. The recruitment of juvenile *T. pavo* in Ischia (Naples, Italy) occurred during the same period, with peaks of sightings in mid-September [43]. Continuous monitoring will be essential in the coming years to determine whether *T. pavo* establishes a stable and growing population in the area. This includes observing additional specimens, if present, and documenting their numbers, size, age classes, and sex over time.

The likely temperature sensitivity of *T. parvo* is exemplified by the high post-settlement mortality rates associated with an unusual drop in water temperature in November 1998 in the Tyrrhenian Sea [42,43]. Recent studies project that Adriatic Sea surface temperatures could increase by approximately 1.5 °C by 2040 (meridionalization), alongside rising sea levels and increased salinity [44,45,46,47]. These environmental changes may facilitate the spread of thermophilic species, such as *T. pavo*, which has been suggested as an indicator of climate change [48]. Other thermophilic species have already been reported in the area, such as the bluefish *Pomatomus saltatrix* (Linnaeus, 1758) [49] and the dinoflagellate *Ostreopsis ovata* [50,51], with *O. ovata* experiencing a local bloom in 2024. Although the long-term establishment of *T. pavo* on the Trabocchi Coast cannot be predicted, continuous yearly monitoring is vital. This report underscores that the discovery of *T. pavo* in the middle Adriatic precedes Milazzo et al.’s [52] forecast, which predicted its presence by 2040, by 16 years.

## 5. Conclusions

Future studies should assess *T. pavo*’s interactions with other species in the area, including competitors such as *Coris julis* (Linnaeus, 1758), which is increasingly common in the region, and at least some species well documented in the area: *Diplodus sargus* (Linnaeus, 1758), *Diplodus vulgaris* (Geoffroy Saint-Hilaire, 1817), *Diplodus puntazzo* (Walbaum, 1792), and *Diplodus annularis* (Linnaeus, 1758), in addition to the sea urchins *Paracentrotus lividus* (Lamarck, 1816), *Arbacia lixula* (Linnaeus, 1758) and *Chromis chromis* (Linnaeus, 1758) [49]. Hence, constant monitoring of the marine environment of the Trabocchi Coast seems essential to quickly figure out the ongoing situation, the trend of the inhabitant species, and their survival. Where required, interventions should be made accordingly and without delay to protect the local biodiversity.

## Data Availability

Data are contained within the article and Supplementary Materials.

## Supplementary Materials

The following supporting information can be downloaded at https://doi.org/10.5281/zenodo.13954778: Video_First record of *T. pavo* in the central Adriatic, Italy.
